# Contemporary epidemiology of gout in the UK general population

**DOI:** 10.1186/ar3272

**Published:** 2011-03-03

**Authors:** Lucía Cea Soriano, Dietrich Rothenbacher, Hyon K Choi, Luis A García Rodríguez

**Affiliations:** 1 Spanish Centre for Pharmacoepidemiologic Research (CEIFE), Almirante 28-2°, 28004, Madrid, Spain; 2 Institute of Epidemiology and Medical Biometry, University of Ulm, Helmholtzstr 22, D-89081, Ulm, Germany; 3 Section of Rheumatology and Clinical Epidemiology Unit, Boston University School of Medicine, 650 Albany Street, Suite 200, Boston, MA 02118,USA

## Abstract

**Introduction:**

The objective of this study was to investigate the contemporary incidence of gout, examine potential risk factors, and evaluate specific gout treatment patterns in the general population.

**Methods:**

Using the health improvement network (THIN) UK primary care database, we estimated the incidence of gout based on 24,768 newly diagnosed gout patients among a cohort of 1,775,505 individuals aged 20 to 89 years between 2000 and 2007. We evaluated potential risk factors for incident gout in a nested case-control study with 50,000 controls frequency-matched by age, sex and calendar time. We calculated odds ratios (OR) by means of unconditional logistic regression adjusting for demographic variables, lifestyle variables, relevant medical conditions and drug exposures.

**Results:**

The incidence of gout per 1,000 person-years was 2.68 (4.42 in men and 1.32 in women) and increased with age. Conventional risk factors were significantly and strongly associated with the risk of gout, with multivariate ORs of 3.00 (95% confidence interval (CI)) for excessive alcohol intake (that is, more than 42 units per week), 2.34 (95% CI 2.22 to 2.47) for obesity (body mass index > = 30 kg/m^2^), 2.48 (95% CI 2.19 to 2.81) for chronic renal impairment, and 3.00 (95% CI 2.85 to 3.15) for current diuretic use. For other medical conditions the multivariate OR were 1.84 (95% CI 1.70 to 2.00) for heart failure, 1.45 (95% CI 1.18 to 1.79) for hypertriglyceridemia and 1.12 (95% CI 1.04 to 1.22) for psoriasis. Use of cyclosporine was associated with an OR of 3.72 (95% CI, 2.17 to 6.40). Among gout-specific therapies, allopurinol was the most frequently used with a one-year cumulative incidence of 28% in a cohort of incident gout diagnosed from 2000 to 2001. Use of gout-specific treatment has not changed over recent years except for an increase of colchicine.

**Conclusions:**

The contemporary incidence of gout in UK remains substantial. In this general population cohort, associations with previously purported risk factors were evident including psoriasis, heart failure, hypertriglyceridemia, and cyclosporine therapy. Use of gout-specific treatment has remained relatively constant in recent years except for an increase of colchicine.

## Introduction

Gout is a common and excruciatingly painful inflammatory arthritis affecting at least 1% of the population in Western countries [1,2]. Gout results from the deposition of urate (monosodium urate monohydrate) crystals in a joint, leading to an acute inflammatory response, or deposition in soft tissues, which can form tophi. Hyperuricemia is considered the precursor of gout, while alcohol intake, obesity, meat and seafood consumption are among the established life-style-related risk factors [3,4]. Factors reducing renal clearance of uric acid such as use of diuretics, and comorbid conditions such as hypertension, and chronic renal impairment are also involved in the pathogenesis of gout [3]. Pharmacologic interventions remain an important part of gout management [5,6].

Several studies have suggested an increase in the disease burden of gout [1,4,7-10]; however, recent data since the new millennium are lacking so far. The aims of this study were to estimate the contemporary incidence of gout in a UK primary care setting, to quantify the magnitude of associations with potential risk factors, including relevant medical conditions and drug exposures, and to describe recent treatment trends in incident gout patients.

## Materials and methods

A nested case-control study was conducted within an assembled cohort in the Health Improvement Network (THIN) database. THIN contains computerized information entered by general practitioners in the UK [11]. Data on about 3,000,000 patients are systematically recorded and sent anonymously to THIN. THIN collects and organizes this information in order for it to be used for research projects. The computerized information includes demographics, details from general practitioners' (GPs) visits, diagnoses from specialists' referrals and hospital admissions, results of laboratory tests and a free text section (information available on request). Prescriptions issued by general practitioners are directly generated from the computer. An additional requirement for participating practices is recording of the indication for new courses of therapy. The Read classification is used to code specific diagnoses, and a drug dictionary based on data from the Multilex classification is used to code drugs [12,13]. The study was approved by a UK Multicentre Research Ethics Committee.

The study population included all individuals aged between 20 to 89 years with a registration status of permanent or died in THIN between January 2000 and December 2007. They were required to have at least two years of enrolment with the GP, and at least one GP visit and one prescription in the two years before entering the source population (start date). This was done to ensure a minimum complete source of information equal to or greater than this time interval prior to entering the study cohort. Individuals with cancer before the start date were excluded. For the ascertainment of incident gout patients, we also excluded all individuals with a gout diagnosis before the start date (considered as prevalent gout) from the study population. All individuals contributed person-time on their respective start date until the earliest of one of the following criteria, whichever came first: code suggesting gout, death, 31 December 2007 or 90^th ^birthday. Our final study population consisted of 1,775,505 individuals followed up an average of 5.2 years.

### Ascertainment of gout patients

The initial ascertainment of gout patients was performed with an automatic computer search. We identified all individuals with a first-ever diagnosis of gout recorded in the database during the study period. Subjects with any prescription of specific gout-treatment (allopurinol, colchicine and uricosuric drugs) before the start date were identified and considered as prevalent gout patients. Finally, we ascertained 24,768 newly diagnosed gout patients (incident gout). The date of gout diagnosis (index date) was defined as the earliest date of first diagnosis of gout or first specific gout-treatment (allopurinol, colchicine and uricosuric drugs) among individuals with a diagnosis of gout. NSAIDs as well as steroids were not included in our case definition to have little specificity to be a treatment for gout and would, therefore, introduce substantial gout misclassification [1]. All incident gout patients were used as cases in the nested case-control analysis.

To evaluate the robustness of gout ascertainment, we performed a sensitivity analysis restricting gout cases to those receiving anti-gout treatment (*N *= 19,749). To this end, we used the following operational definition: we identified within 90 days after the first-ever diagnosis of gout (index date) any anti-gout treatment (colchicine, probenecid and uricosuric drugs) and/or a prescription of NSAIDs on the same index date. A similar case definition of gout has been shown to have a validity of 90% in the General Practice Research Database (GPRD) [14,15].

### Selection of controls

We randomly selected 50,000 controls using density sampling. This was done by generating at random a date encompassed within the study period for each of the members of the study population. If the random date for a study member was included in his/her eligible person-time (follow-up period), we marked that person-day as an eligible control. The same exclusion criteria were applied to controls as to cases. Controls were frequency-matched to cases by age within one year, sex and calendar year (year of newly diagnosed gout). The random date for controls was used as an index date in the nested case-control analysis.

### Exposure assessment

We collected from the database all the information any time prior to the index date for traditional life style factors: smoking, alcohol intake (units per week), personal characteristics such as body mass index (BMI) in kg/m^2^, as well as comorbidities such as ischemic heart diseases, hypertension, hyperlipidemia, chronic renal impairment and other disorders. In addition, the number of GP visits, referrals, and hospitalizations were collected in the year prior to index date. We classified exposure of drugs into four mutually exclusive time windows: *current use *(recent prescription lasted until index date or ended in the 30 days prior); *recent *(finished between 31 and 365 days), *past *(finished more than 365 days), and *never use *(no recorded use at any time prior to index date). Duration of treatment was calculated among current users of drugs and was computed summing the time of consecutive prescriptions (allowing for a free interval gap no greater than 60 days, complete adherence).

### Statistical analysis

Incidence rates of gout over the whole study period as well as age- and sex-specific ones were estimated and 95% confidence intervals were calculated. We also computed incidence at the beginning (2000 to 2001) and end (2006 to 2007) of the study period to analyze potential secular trends.

A nested case-control analysis was performed to estimate the odds ratios (OR) and 95% confidence intervals (CI) of gout associated with traditional life-style factors, relevant medical conditions and drug exposures by means of unconditional logistic regression. The multivariable model included the frequency-matched variables, as well as the number of general practitioner (GP) visits, and other established risk factors such as alcohol consumption, body mass index (BMI), ischemic heart disease (IHD), hypertension, hyperlipidemia, diabetes, chronic renal impairment, and diuretic use.

We analyzed gout treatment patterns according to time since the first appearance of a gout diagnosis among gout patients. We examined the following drug treatments separately: allopurinol, colchicine and uricosuric drugs (which included probenecid and sulfinpyrazone). We estimated the cumulative incidence of the specific gout treatment (defined as receiving at least one prescription) in our incident gout population. We did not include NSAIDs or steroids as they have multiple other indications and are less specifically indicated for gout. For our treatment pattern analysis, we restricted our cohort of incident gout to patients diagnosed in 2000 and 2001 to ensure the longest possible length of disease duration. In this inception cohort, we studied treatment patterns over five time intervals: at one year, three years, five years, seven years and nine years after the first appearance of a gout diagnosis. We also compared the cumulative incidence of anti-gout treatment at one year after gout onset between patients who developed incident gout in 2000 to 2001 and those in 2006 to 2007. All statistical procedures were performed with the STATA package version 11.0 (StataCorp LP, College Station, TX, USA).

## Results

**Figure 1:**
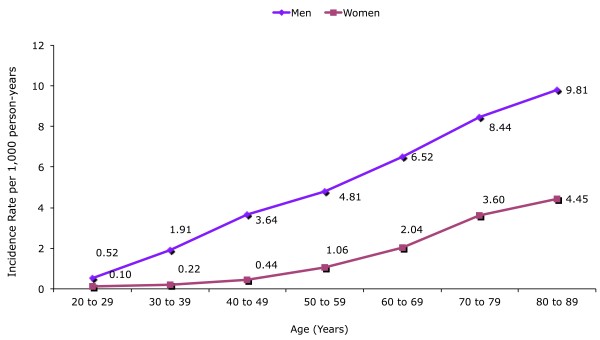
**Sex and age specific incidence rate of gout 2000 to 2007 in the THIN database**. Incidence rates per 1,000 person-years: overall 2.68 (95% CI 2.65 to 2.72), men 4.42 (95% CI 4.36 to 4.48), women 1.32 (95% CI 1.29 to 1.35)

We identified 24,768 newly diagnosed gout patients corresponding to a crude incidence rate of 2.68 (95% CI 2.65 to 2.72) per 1,000 person-years among individuals aged 20 years or older. The incidence of gout was 4.42 (95% CI 4.36 to 4.48) in men and 1.32 (95% CI 1.29 to 1.35) in women per 1,000 person-years. The incidence of gout increased with increasing age (Figure 1). The corresponding male/female ratio was 3.4. When we restricted our population to individuals aged 70 years and above the corresponding male/female ratio was 2.3. Mean age at first diagnosis of gout was 60.1 years (95% CI 59.9 to 60.4) among men and 67.7 years (95% CI 67.3 to 68.0) among women, respectively.

**Figure 2:**
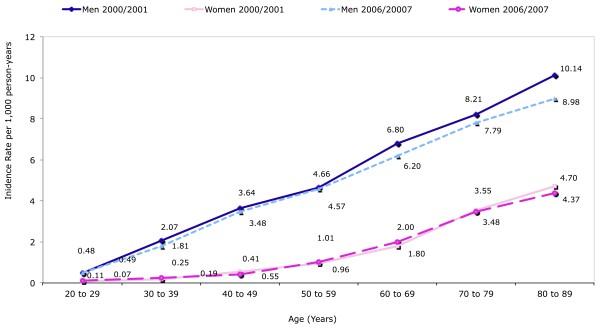
**Sex and age specific incidence rate of gout in 2000/2001 and 2006/2007**. 2000 to 2001: Incidence rates per 1,000 person-years: overall 2.67 (95% CI 2.59 to 2.75), men 4.48 (95% CI 4.33 to 4.64), women 1.28 (95% CI 1.21 to 1.35). 2006 to 2007: Incidence rates per 1,000 person-years: overall 2.52 (95% CI 2.46 to 2.57), men 4.01 (95% CI 3.91 to 4.12), women 1.25 (95% CI 1.20 to 1.30).

Incidence rate was 2.67 (95% CI 2.59 to 2.75) in 2000 to 2001 and 2.52 (95% CI 2.46 to 2.57) in 2006 to 2007. Sex and age specific incidence rate remained relatively constant between both time periods (Figure 2).

Table 1 shows the results of the nested case-control analysis. Among the traditional risk factors for gout, obesity, alcohol consumption, diuretics use, and hypertension were independently associated with the risk of incident gout. A dose response was observed according to alcohol consumption and BMI. Consumers between 25 and 42 units per week presented an OR of 2.45 (95% CI 2.27 to 2.63), and 3.00 (95% CI 2.66 to 3.38) among consumers greater than 42 units per week compared with abstainers. Individuals with a BMI between 25 and 29 kg/m^2 ^had an OR of 1.62 (95% CI 1.55 to 1.70) and those with BMI of 30 kg/m^2 ^or greater an OR of 2.34 (95% CI 2.22 to 2.47) compared to those with a BMI in the normal range (20 to 24 kg/m^2^). A history of chronic renal impairment showed an OR of 2.48 (95% CI 2.19 to 2.81) and hypertension an OR of 1.18 (95% CI 1.13 to 1.23).

**Table 1:** Relative risk (OR) of gout according to lifestyle factors and comorbidities

Characteristics	Controls *N *= 50,000 N (%)	Gout cases *N *= 24,768 N (%)	Multivariate OR (95% CI)*
Sex			
Male	36,953 (73.91)	17,946 (72.46)	-
Female	13,047 (26.09)	6,822 (27.54)	-
Age			
20 to 49 years	10,211 (20,42)	5,290 (21.36)	-
50 to 59 years	10,111 (20.22)	4,873 (19.67)	-
60 to 69 years	11,930 (23.86)	5,753 (23.23)	-
70 to 79 years	11,896 (23.79)	5,858 (23.65)	-
80 to 89 years	5,852 (11.70)	2,994 (12.09)	-
Alcohol (units per week)‡			
Non-use	16,396 (32.79)	7,628 (30.80)	1 (ref)
1 to 9 u/w	13,362 (26.72)	5,960 (24.06)	1.06 (1.01 to 1.11)
10 to 24 u/w	8,150 (16.30)	5,074 (20.49)	1.56 (1.49 to 1.65)
25 to 42 u/w	2,152 (4.30)	2,053 (8.29)	2.45 (2.27 to 2.63)
more than 42 u/w	633 (1.27)	739 (2.98)	3.00 (2.66 to 3.38)
unknown	9,307 (18.61)	3,314 (13.38)	1.15 (1.08 to 1.23)
BMI (kg/m^2^)			
15 to 19	1,588 (3.18)	315 (1.27)	0.66 (0.58 to 0.76)
20 to 24	13,899 (27.80)	4,225 (17.06)	1 (ref)
25 to 29	16,623 (33.25)	9,169 (37.02)	1.62 (1.55 to 1.70)
> = 30	7,412 (14.82)	7,176 (28.97)	2.34 (2.22 to 2.47)
unknown	10,478 (20.96)	3,883 (15.68)	1.48 (1.39 to 1.57)
Number of GP visits			
0 to 4 visits	22,700 (45.40)	6,785 (27.39)	1 (ref)
5 to 9 visits	13,201 (26.40)	6,692 (27.02)	1.38 (1.32 to 1.44)
10 to 19 visits	10,422 (20.84)	7,400 (29.88)	1.66 (1.58 to 1.74)
> = 20 visits	3,677 (7.35)	3,891 (15.71)	2.13 (1.99 to 2.27)
Chronic renal failure	467 (0.93)	947 (3.82)	2.48 (2.19 to 2.81)
Hypertension	16,280 (32.56)	12,858 (51.91)	1.18 (1.13 to 1.23)
Heart failure	1,422 (2.84)	2,142 (8.65)	1.84 (1.70 to 2.00)
Ischemic heart disease	6,716 (13.43)	4,923 (19.88)	1.19 (1.14 to 1.25)
Hyperlipidemia			
Hypercholesterolemia†	4,188 (8.38)	3,124 (12.61)	1.08 (1.02 to 1.14)
Hypertrygliceridemia†	218 (0.44)	216 (0.87)	1.45 (1.18 to 1.79)
Other dyslipidemia†	1,849 (3.70)	1,699 (6.86)	1.21 (1.12 to 1.31)
Diabetes	3,885 (7.77)	2,404 (9.71)	0.66 (0.62 to 0.71)
Nephrolithiasis	776 (1.55)	446 (1.80)	1.03 (0.91 to 1.18)
Psoriasis	1,894 (3.79)	1,173(4.74)	1.12 (1.04 to 1.22)

*Odds ratio (OR) adjusted for sex, age, calendar year, number of GP visits, BMI, alcohol consumption, IHD, hypertension, hyperlipidemia, diabetes, chronic renal failure and use of diuretics.

†Note: The estimate of each variable was calculated by removing hyperlipidemia from the model.

‡1 unit = 10 ml of pure ethanol (eight grams of alcohol)

Among suspected medical conditions, prior history of congestive heart failure was strongly associated with an increased risk of incident gout, whereas ischemic heart disease was modestly associated with an increased risk. While hypertriglyceridemia was associated with a clear increased risk of gout (multivariate OR, 1.45 (95% CI, 1.18 to 1.79), other dyslipidemias and hypercholesterolemia presented an OR of 1.21 (95% CI, 1.12 to 1.31), and 1.08 (95% CI, 1.02 to 1.14), respectively. No increased risk was found among patients with prior nephrolithiasis, whereas psoriasis was associated with a slightly increased risk of gout (multivariate OR, 1.12; 95% CI, 1.04 to 1.22). The only medical condition associated with a reduced risk of gout was diabetes with an OR of 0.66 (95% CI 0.62 to 0.71) (Table 1).

Both current and recent users of diuretics showed a significantly higher risk of gout. This increased risk returned to the baseline one year after stopping diuretics (Table 2). Additional analyses showed that after adjusting for heart failure, the increased risk among current users of diuretics was slightly reduced, but remained strong (OR (2.76: 95% CI 2.62 to 2.90)). The risk of gout was 2.20 (95% CI 2.05 to 2.38) among diuretic current users with less than one-year duration. The estimates were 2.85 (95% CI 2.62 to 3.11) and 3.42 (95% CI 3.23 to 3.62) among users on diuretics treatment between one to two years and longer than two years, respectively (*P *for duration response <0.001).

**Table 2:** Relative risk (OR) of gout associated with use of various medications

Co-medication	Controls *N *= 50,000 N (%)	Gout Cases *N *= 24,768 N (%)	Multivariate OR (95%CI)*
Diuretics			
Never use	36,237 (72.47)	12,206 (49.28)	1(ref)
Current (<31 days)	8,582 (17.16)	9,921 (40.06)	3.00 (2.85 to 3.15)
Recent (31 to 365 days)	1,462 (2.92)	1,050 (4.24)	1.83 (1.67 to 2.01)
Past (>365 days)	3,719 (7.44)	1,591 (6.42)	1.10 (1.02 to 1.18)
Aspirin low-dose			
Never use	38,737 (77.47)	16,924 (68.33)	1 (ref)
Current (<31 days)	7,788 (15.58)	5,291 (21.36)	0.95 (0.90 to 1.00)
Recent (31 to 365 days)	1,292 (2.58)	938 (3.79)	1.12 (1.02 to 1.24)
Past (>365 days)	2,183 (4.37)	1,615 (6.52)	1.10 (1.02 to 1.19)
NSAIDs			
Never use	20,989 (41.98)	6,735 (27.19)	1 (ref)
Current (<31 days)	4,064 (8.13)	4,791 (19.34)	3.07 (2.90 to 3.24)
Recent (31 to 365 days)	5,616 (11.23)	4,107 (16.58)	2.05 (1.94 to 2.16)
Past (>365 days)	19,331 (38.66)	9,135 (36.88)	1.29 (1.24 to 1.35)
Transplant rejection drugs†			
Never use	49,792 (99.58)	24,586 (99.27)	1 (ref)
Current (<31 days)	94 (0.19)	90 (0.36)	1.12 (0.80 to 1.57)
Recent (31 to 365 days)	21 (0.04)	23 (0.09)	1.50 (0.77 to 2.93)
Past (>365 days)	93 (0.19)	69 (0.28)	1.14 (0.81 to 1.60)
Azathioprine			
Never use	49,794 (99.59)	24607 (99.35)	1 (ref)
Current (<31 days)	89 (0.18)	65 (0.26)	0.93 (0.64 to 1.34)
Recent (31 to 365 days)	22 (0.04)	20 (0.08)	1.11 (0.56 to 2.22)
Past (>365 days)	95 (0.19)	76 (0.31)	1.21 (0.87 to 1.68)
Cyclosporine			
Never use	49,941 (99.88)	24,657 (99.55)	1 (ref)
Current (<31 days)	20 (0.04)	75 (0.30)	3.72 (2.17 to 6.40)
Recent (31 to 365 days)	6 (0.01)	10 (0.04)	1.42 (0.46 to 4.38)
Past (>365 days)	33 (0.07)	26 (0.10)	0.91 (0.52 to 1.61)
Hormonal replacement therapy (HRT) *(women only)*			
Never use	10,436 (79.99)	5,368 (78.69)	1 (ref)
Current (<31 days)	785 (6.02)	345 (5.06)	0.86 (0.74 to 1.01)
Recent (31 to 365 days)	347 (2.66)	192 (2.81)	1.07 (0.87 to 1.32)
Past (>365 days)	1,479 (11.34)	917 (13.44)	1.02 (0.91 to 1.13)

*Odds ratio (OR) adjusted for sex, age, calendar year, number of GP visits, BMI, alcohol consumption, IHD, hypertension, hyperlipidemia, diabetes, chronic renal failure and use of diuretics.

† Transplant rejection drugs include: azathioprine, mycophenolate mofetil, mycophenolic acid, sirolimus, tacrolimus.

NSAIDs, nonsteroidal anti-inflammatory drugs

Among suspected medications, current users of low dose aspirin presented a multivariate OR of 0.95 (95% CI 0.90 to 1.00). When we analyzed the association with aspirin < = 75 mg daily (mini-dose aspirin), which was the dose used in 80% of aspirin users, the estimate of risk remained the same (0.95, 95% CI 0.90 to 1.00). NSAID current users showed a multivariate OR of 3.07 (95% CI 2.90 to 3.24) (Table 2).

When we also analyzed the association according to the time when NSAID treatment was started, it was apparent that the risk was mainly concentrated among current users within the first month with an OR of 7.01 (95% CI 6.46 to 7.61) while those with a use of one year or greater presented an OR of 1.11 (95% CI 1.01 to 1.23), suggesting reverse causality (that is, potential NSAID: treatment for early gout symptoms), among users in the first month of use (data not shown).

Use of cyclosporine was rare, but was associated with an OR of 3.72 (95% CI 2.17 to 6.40). The risk was rather constant over treatment duration with an OR of 4.16 (95% CI 2.21 to 7.83) among users of one year or longer. Azathioprine, another post-organ transplantation drug, showed an OR of 0.93 (95% CI 0.64 to 1.34). We found women on hormone replacement therapy (HRT) to have an OR of 0.86 (95% CI 0.74 to 1.01).

**Figure 3:**
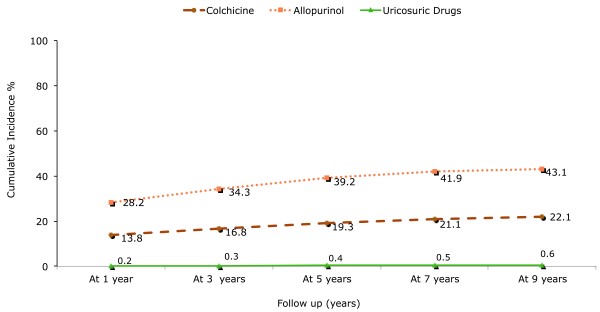
**Cumulative incidence of gout treatment over gout disease duration**.

Next, we analyzed gout treatment patterns during recent years. Cumulative incidence of specific gout treatment at different time intervals among the subcohort of newly diagnosed gout patients in 2000 to 2001 (*N *= 4,349) is presented in Figure 3. One year after first-ever gout diagnosis, use of allopurinol presented the highest cumulative incidence with 28.2% (95% CI, 26.9 to 29.6) followed by colchicine with 13.8% (95% CI 12.8 to 14.8). Use of uricosuric agents was very low with a cumulative incidence of 0.2% (95% CI, 0.1 to 0.4). At nine years, the cumulative incidence of the various gout treatments was about twice as high compared to the use during the first year after gout diagnosis. This pattern was consistent among the three anti-gout treatments.

**Figure 4:**
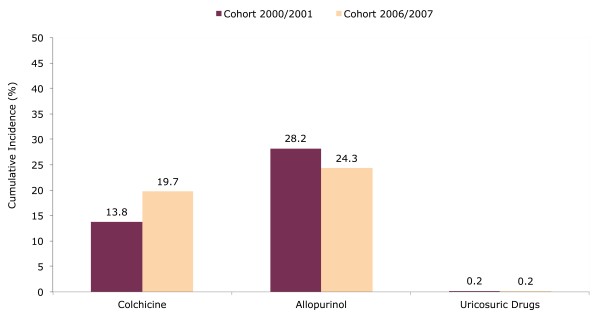
**One-year cumulative incidence of gout treatment in 2000 to 2001 and 2006 to 2007**.

When we analyzed the use during the first year in a cohort of gout diagnosed in 2006 to 2007 (*N *= 7,089), the cumulative incidence was 24.3% (95% CI 23.3 to 25.3) for allopurinol and 0.2% (95% CI 0.1 to 0.3) for uricosuric drugs. Only colchicine showed a higher use in 2006 to 2007 with a cumulative incidence of 19.7% (95% CI 18.8 to 20.7) compared to 13.8 (95% CI 12.8 to 14.8) in 2000 and 2001 (Figure 4).

## Discussion

In this large UK primary care population, we found that the incidence of gout remained stable between 2000 and 2007. Our contemporary data corroborated previously shown risk factors, including alcohol intake, obesity, chronic renal impairment, cardiovascular conditions, and diuretic use. Furthermore, we have examined other purported medical conditions and medications, about which we are not aware of any previous large-scale evidence. We found that prior histories of ischemic heart disease, heart failure, hyperlipidemia, and psoriasis are independently associated with an increased risk of gout, whereas diabetes was associated with a lower risk of gout. Interestingly, among subtypes of hyperlipidemia, hypertriglyceridemia was associated with an increased risk, whereas this pattern was much less clear among patients with hypercholesterolemia. We also found that among post-transplant drugs cyclosporine was the only agent associated with increased risk of gout. Finally, the frequency of anti-gout medication use has remained relatively unchanged in recent years except for an increase of colchicine.

Our estimate of incidence rate increased from 0.4 per 1,000 person-years in women of 40 to 49 years, to 3.6 in women of 70 to 79 years. Our results are in line with a prior study [16] that reported an incidence rate of gout of 0.6 per 1,000 person-years in women <45 years and 2.5 in women over the age of 75. When we restricted our cohort to individuals aged 40 years and older, our estimate of incidence (3.5 per 1,000 person-years) was in close agreement with a recent UK study, which estimated an incidence rate of gout 3.2 per 1,000 person-years [9]. Although the mean age of first diagnosis was 60.1 years in males and 67.7 years in females, almost 40% of all incident gout cases were younger than 60 years, indicating that many middle-aged subjects were affected. Male preponderance was more marked in individuals less than 60 years. The different age dependence between males and females in the incidence of gout could in part be due to hormonal status. The uricosuric effects of estrogens could lead to a protective effect on the risk of gout in premenopausal women [17-20]. We also observed that women on exogenous hormonal treatment had 14% less risk of developing gout, and the reduced risk disappeared after stopping HRT, a finding previously reported in a large prospective cohort study of female nurses [16].

Our findings confirm the relationship between traditional risk factors with well-known effects on increasing uric acid levels and subsequently the risk of gout [21,22]. Our cohort data showed a clear dose-response relation between alcohol consumption and the risk of gout, similar to previous studies [23]. Similarly, our results showed a continuous increased risk of gout with increasing BMI as well as a protective effect in individuals with a BMI under 20 kg/m^2^. Bidirectional associations between weight loss and the prevention of gout and weight gain and the development of gout were reported in the Health Professionals Follow up Study [10]. Chronic renal impairment more than doubled the risk of developing incident gout, which is likely due to decreased urate excretion resulting in uric acid accumulation [4,24]. Beyond the effect of this and other medical conditions, the use of diuretics, in particular among users of one year and longer than one year, was associated with a three-fold increased risk of gout.

We also evaluated other suspected risk factors lacking in large-scale epidemiologic data, such as lipid abnormalities, ischemic heart disease, congestive heart failure, psoriasis, and various medications. While all subtypes of hyperlipidemia showed independent associations with the risk of incident gout [25], the association with hypertriglyceridemia was most prominent. Elevated triglyceride level is a cardinal feature of insulin resistance, which is closely associated with elevated serum uric acid levels. Individuals with diabetes showed a reduced risk of gout. Some studies have reported lower uric acid levels among diabetes patients [26,27]. We also found that prior history of congestive heart failure and ischemic heart disease was independently associated with an increased risk of gout. Associated relative tissue hypoxia, increased lactate levels, or accelerated adenosine triphosphate (ATP) consumption could increase the risk of hyperuricemia and gout in patients with these conditions [28,29]. Recently, a case-control study with only 60 individuals with gout and 6 with heart failure (HF) reported an increased risk of gout among patients with HF [30,31]. Interestingly, this study also reported that diuretic use did not increase the risk of gout after adjusting for HF and other cardiovascular conditions, as these factors are closely associated with each other [30,31]. Our study, with substantially larger study samples (n with gout = 24,768 and n with HF = 3,564), was able to detect independent associations with each of these two factors. Psoriasis is a disorder associated with increased cell turnover leading to increased uric acid production [3] and our cohort confirmed a small but significantly increased risk of incident gout. Finally, the absence of an association between history of nephrolithiasis and risk of gout was consistent with the result reported by Kramer *et al*. [32,33].

Current use of low-dose aspirin was more common among gout patients than controls. However, after adjusting for other covariates, including history of ischemic heart disease, low-dose aspirin was not associated with an increased risk of gout. This suggests that the independent pathogenetic role of low-dose aspirin use, if any, may be minor on the risk of incident gout. Our study confirmed an increased risk of gout among users of cyclosporine (an immunosuppressant drug indicated in organ transplant, psoriasis, rheumatoid arthritis, and nephrotic syndrome), whereas there was no association with use of azathioprine or other medications indicated after organ transplant [4,34,35]. The apparent association between NSAID use and risk of gout was restricted to patients recently started before the recorded diagnosis of gout. This observation suggests substantial confounding by indication, where use within the first month could be a proxy (treatment) for early manifestations of gout, although a true association cannot be ruled out entirely.

In terms of anti-gout medication use during recent years, we found that allopurinol use had the highest frequency, followed by colchicine, whereas a small proportion of gout patients were taking uricosuric drugs. Treatment patterns did not change in recent years, with only colchicine showing an increase. While allopurinol use seems to be in line with a previous study based on the GPRD between 1990 and 1999, colchicine use appears to have increased since the 1990s, when the authors reported an annual frequency of colchicine use of only 1 to 3% [1]. In addition, it should be noted that the greater proportion of colchicine use could also be explained in part by the fact that our study is based exclusively on individuals newly-diagnosed with a first gout attack, when colchicine prophylaxis is used more often [6].

The strengths and limitations of our study deserve comment. Our study was performed with a large population-based database which contains computerized information entered by primary care physicians that permits the extrapolation of results to the general population. Also, under our design of incidence density sampling, the OR is an unbiased estimator of the incidence rate ratio. Some level of misclassification is unavoidable when working with computerized databases; however, the impact of non-differential misclassification would have most likely biased our estimates of effect toward the null and would not explain the strong associations and dose-response relationships observed in our study. Furthermore, in a secondary analysis when we restricted gout cases to those with GPs' diagnoses of gout combined with anti-gout medication use, our results remained similar (data not shown).

## Conclusions

In conclusion, our findings suggest that the contemporary disease burden of gout remains substantial in the UK. Previously identified risk factors still pose considerable relative risk in this cohort, including alcohol intake, obesity, chronic renal impairment, several cardiovascular conditions, and diuretic use. In addition, we found independent associations with prior history of ischemic heart disease, hyperlipidemia, heart failure and psoriasis, and with the use of cyclosporine. These data support the notion that appropriate pharmacologic management and control of lifestyle factors such as maintenance of a healthy BMI, alcohol consumption, and comorbidities, including hypertension and chronic renal failure, would reduce the burden of this common and excruciatingly painful inflammatory arthritis.

## Abbreviations

ATP: adenosine triphosphate; BMI: body mass index; CEIFE: Spanish Centre for Pharmacoepidemiologic Research; CI: confidence interval; GP: general practitioner; GPRD: general practice research database; HF: heart failure; HRT: hormonal replacement therapy; IHD: ischemic heart disease; NSAID: non-steroidal anti-inflammatory drug; OR: odds ratio; THIN: The Health Improvement Network.

## Competing interests

Lucía Cea-Soriano and Dr García Rodríguez work for CEIFE, which has received unrestricted research grants from Novartis Pharma AG. Dr. Choi received research funding for other projects from Takeda Pharmaceuticals and served on advisory boards for Takeda Pharmaceuticals and Savient Pharmaceuticals. Dr Rothenbacher was an employee of Novartis Pharma AG until November 2010.

## Authors' contributions

LCS contributed to study design, data collection, statistical analysis, interpretation of data and drafting the manuscript. DR contributed to study design, interpretation of data and reviewed the manuscript. HKC contributed to interpretation of data and statistical analysis and reviewed the manuscript. LAGR contributed to study design, statistical analysis, interpretation of data and reviewed the manuscript. All the authors agreed to the final approval of the version of the article to be published.
